# Treatment of Human Urinary Kallidinogenase Combined with Maixuekang Capsule Promotes Good Functional Outcome in Ischemic Stroke

**DOI:** 10.3389/fphys.2018.00084

**Published:** 2018-02-12

**Authors:** Juexian Song, Yi Lyu, Miaomiao Wang, Jing Zhang, Li Gao, Xiaolin Tong

**Affiliations:** ^1^Department of Neurology, Xuanwu Hospital, Capital Medical University, Beijing, China; ^2^Department of Medical Affairs, Techpool Biopharma Co., Ltd., Guangzhou, China; ^3^Guang'anmen Hospital, China Academy of Chinese Medical Science, Beijing, China

**Keywords:** human urinary kallidinogenase, Maixuekang capsule, acute ischemic stroke, vascular disease, integrative medicine

## Abstract

**Aims:** To evaluate the clinical efficacy of Human Urinary Kallidinogenase (HUK) and Maixuekang capsule in the treatment of acute ischemic stroke (AIS) patients.

**Methods:** In this study, from January 2016 to July 2016, 60 patients with acute ischemic stroke were enrolled and 56 patients with complete information of whom 21 patients received HUK+ basic treatment (HUK group), 16 patients received HUK+ Maixuekang capsule + basic treatment (HUK+ Maixuekang group), 19 patients received basic treatment (control group). 0.15 PNA unit of HUK injection plus 100 ml saline in intravenous infusion was performed in the HUK group and HUK+ Maixuekang group, with once a day for 14 consecutive days. 0.75 g Maixuekang capsules were taken in HUK+ Maixuekang group, with three times a day for 14 consecutive days. The National Institutes of Health Stroke Scale (NIHSS) scores in three groups were analyzed 7 days after treatment. The modified Rankin Scale (mRS) scores in three groups were analyzed 12 month after the treatment.

**Results:** No difference was found in the NIHSS scores, age, gender, and comorbidities between three groups before treatment (*p* > 0.05). Seven days after treatment, the NIHSS scores in the HUK group and HUK+ Maixuekang group were significantly decreased than before (p _HUK_ = 0.001, p _HUK+Maixuekang_ < 0.001), and lower than that in the control group (p _HUK_ = 0.032; p _HUK+Maixuekang_ < 0.001). Twelve months after treatment, good functional outcome rate (12 month mRS score ≤ 2) in the HUK group and HUK+ Maixuekang group was significantly higher than that in the control group (p _HUK_ = 0.049, p _HUK+Maixuekang_ = 0.032).

**Conclusion:** The treatment of HUK or HUK combined with Maixuekang capsule can effectively improve the neurological function and promote long-term recovery for AIS patients.

## Introduction

Stoke is the second most common cause of death and the leading cause of adult disability worldwide (Bonita et al., 2004). Acute cerebral infarction (ACI) is caused by severely reduced blood and oxygen supply (Miao et al., 2016). The clinically-validated treatment for stroke now available is acute thrombolysis, and the utility of this approach is constrained by the need to initiate treatment within 4.5 h of symptoms and the risk of causing cerebral hemorrhage. These factors have limited the number of stroke patients receiving this treatment to <5%, even in areas where this treatment modality is readily available (Chen et al., 2014). Therefore, new therapies for AIS is still in urgent need.

The human urinary kallidinogenase (HUK), a glycoprotein extracted from male urine, can regulate kallikreinkinin system (KKS) by producing kallikrein to promote angiogenesis, enhance cerebral perfusion, and suppress the inflammatory response which has been proven in animal studies (Han et al., 2015). Previous studies have shown that the hirudin, which is the main component of Maixuekang capsule has the ability of protecting the vascular endothelial function, anticoagulation, antiplatelet, regulating blood lipid, alleviating vasospasm, and reducing inflammation (Ge et al., 2014). Therefore, this study aimed to assess the effects of HUK and Maixuekang capsule on prognosis in AIS patients.

## Materials and methods

### Patients

From January 2016 to July 2016, 60 consecutive AIS patients, who were admitted in the neurology department of our hospital, were enrolled in this study. All patients signed informed consent before treatment. They were in accordance with the diagnostic criteria of cerebral infarction approved by the fourth national cerebrovascular academic conference (1995). Inclusion criteria: (1) age from 18 to 90 years; (2) patients with the first onset of stroke; (3) onset time less than 72 h; (4) stroke confirmed by head computed tomography (CT, Siemens SO—MATOM Definition Flash CT, parameters: 120 kV, 150 mAs, thickness 5 mm, interval 5 mm) scan or magnetic resonance imaging (MRI, Siemens Avanto 1.5T MR, parameters: DWI: TE or TR: 102 or 10,000 ms); (5) patients without incomplete hepatic and renal function; (6) patients without severe infectious disease; (7) patients without severe psychotic disease, including dementia; (8) patients without high sensitivity to medicine in this study; (9) patients without history of hemorrhagic stroke, brain tumor, and brain trauma.

### Therapeutic methods

Twenty-two patients were included in HUK group who received 0.15 PNA unit of HUK (Trade name: Kailikang, Guangdong Techpool Bio- Pharma Co., Ltd. with approved medicine of H20052065) injection plus 100 ml saline in intravenous infusion, with once a day for 14 consecutive days. Twenty patients were included in HUK+ Maixuekang group who received not only HUK treatment like HUK group but also 0.75 g Maixuekang capsules, with three times a day for 14 consecutive days. Patients in control group were only given basic treatment (including anti-oxidative therapy and other symptomatic treatment). Other therapeutic drugs on cerebral infarction were forbidden and basic treatment was administrated on all patients.

### Study design

This was a single-center, registry-based, prospective study. All the patients were evaluated by the same group of trained neurologists. Baseline characteristics included gender, age, comorbidities (hypertension and diabetes) and history of smoking and drinking, CHA_2_DS_2−_VASc [congestive heart failure, hypertension, age 75 y (doubled), diabetes mellitus, stroke(doubled)- vascular disease, age 65–74 and sex category(female) score, C reactive protein (CRP) level], and echocardiographic parameters (left atrium dimension, LA; right atrium dimension, RA; left ventricle dimension, LV; right ventricle dimension, RV; left ventricular ejection fraction, LVEF; E-point of septal separation/A-point of septal separation, E/A). National Institute of Health stroke scale (NIHSS) scores were evaluated before treatment and after treatment. The mRS scores at 12 month were obtained by telephone follow-up. Patients' baseline characteristics, NIHSS scores before and after treatment and 12 month good functional outcome (12 month mRS score ≤ 2) rate were compared.

### Study endpoints

The 12 month good functional outcome (12 month mRS score ≤ 2) rate was the primary endpoints. The Gantt chart of this study was display as Figure 2.

### Statistical analyses

The continuous variables fitting the normal distribution were expressed as mean ± standard deviation (SD). The categorical variables were expressed by number and percentage. The student's *t*-test or Fisher's exact test was used for continuous variables and the Chi-squared test was used for categorical variables. Statistical analyses were performed using SPSS20.0 software (IBM SPSS, Armonk, NY, USA) *P* < 0.05 was considered to be statistically significant.

## Results

**Figure 1:**
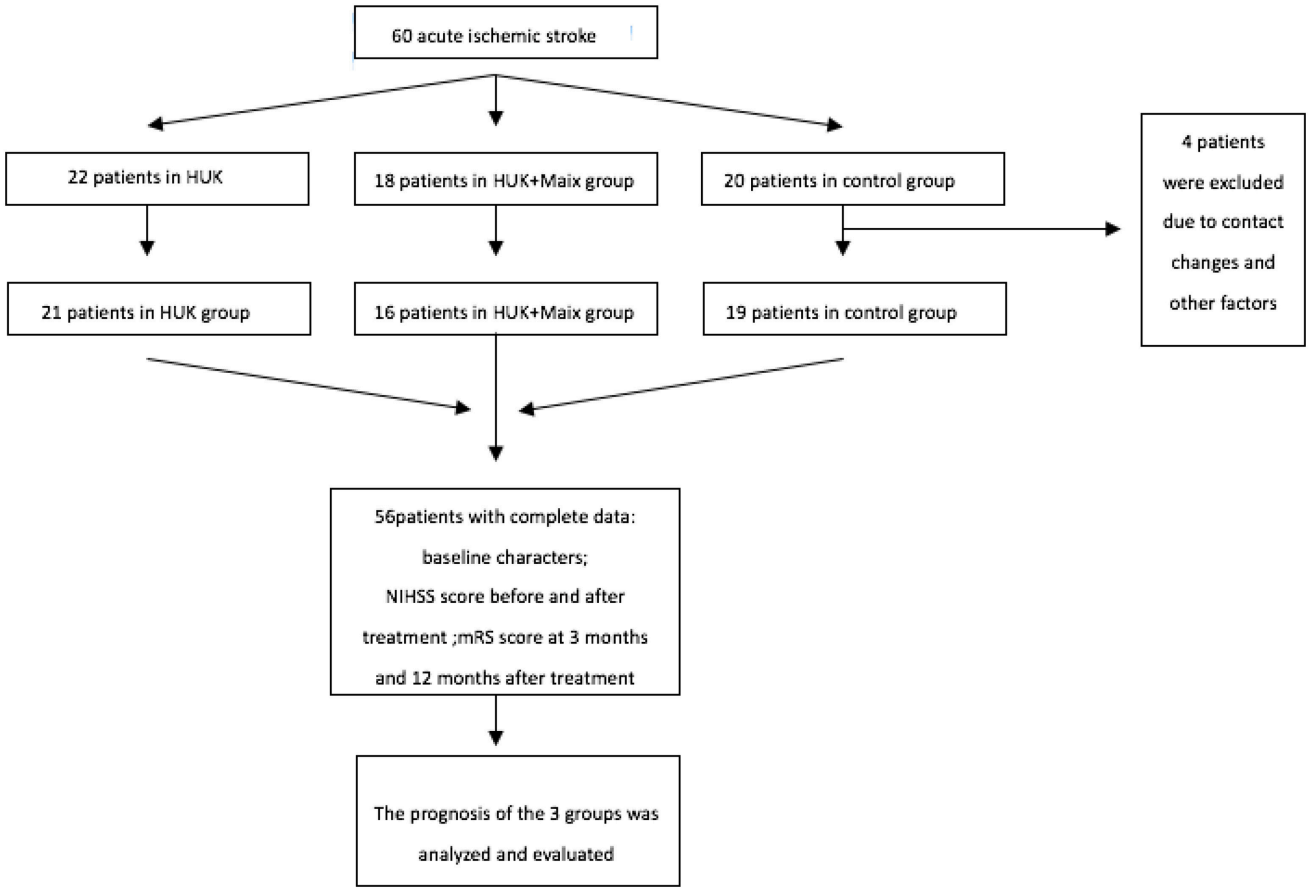
Schematic representation of the study.

This study totally included 60 patients, 4 patients were excluded due to contact changes and other factors. The clinical data and follow-up information of the three groups were analyzed and compared (Figure 1). There was no significance in incidence of acute cerebral events among different groups.

**Figure 2:**
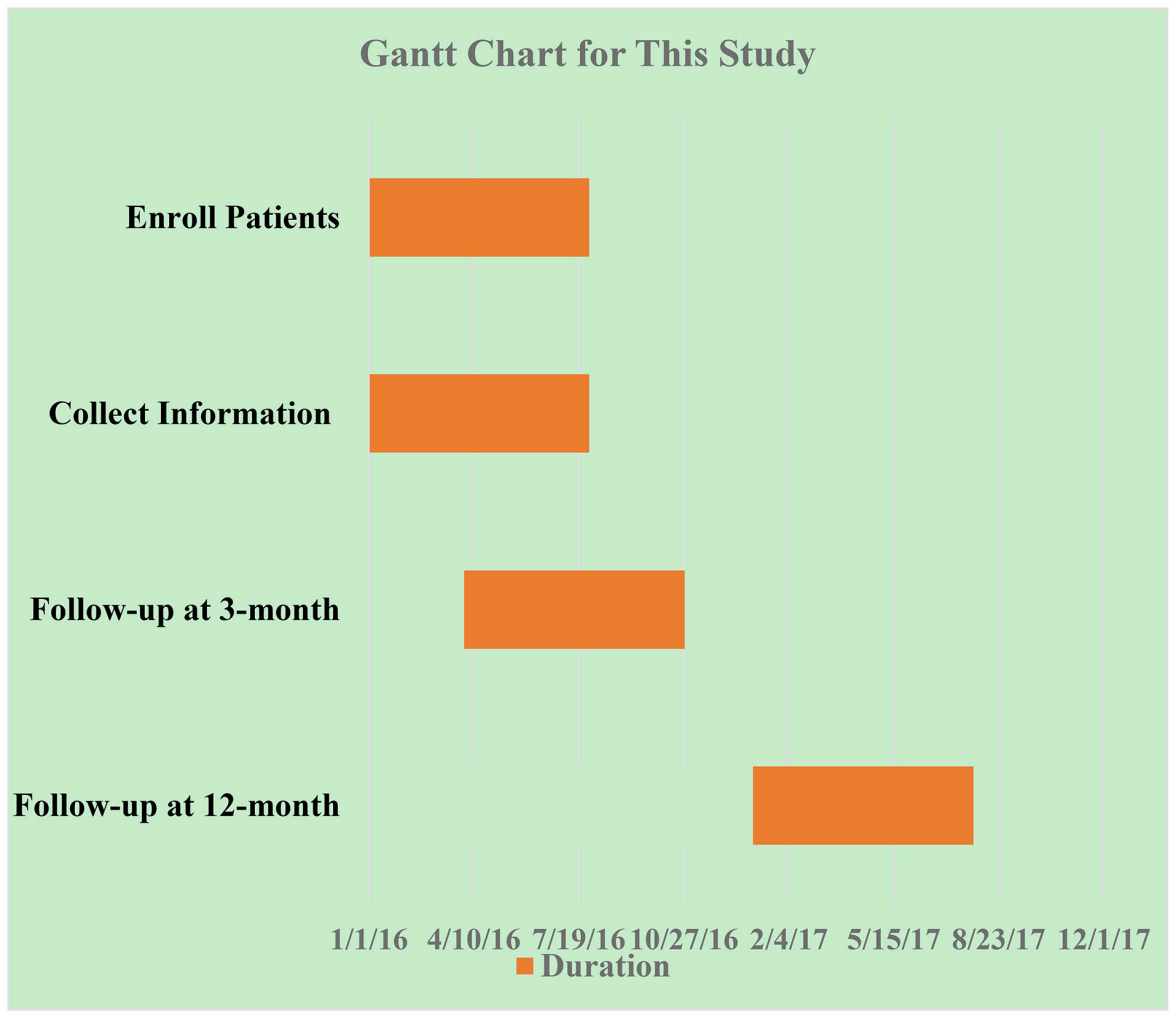
Gantt chart of the study. Enrolled patients: 20160101-20160730; Collect information:20160101-20160730; Follow-up at 3 month: 20160401-20161030; Follow-up at 12 month: 20170101-20170730.

### Baseline characteristics

There were 14 males and 7 females in the HUK group, with an average age at 58.10 ± 13.66 years old and the NIHSS score of this before treatment was 5.19 ± 1.60; There were 11 males and 5 females in the HUK + Maixuekang group, with an average age at 60.88 ± 11.85 years old and the NIHSS score of this before treatment was 5.12 ± 1.75; There were 13 males and 6 females in the control group, with an average age at 61.1 ± 14.46 years old and the NIHSS score of this before treatment was 5.21 ± 1.69; There was no significant difference between these three groups in age, gender, NIHSS score, CHA_2_DS_2−_VASc score, CRP level before treatment and echocardiographic parameters (*p* > 0.05). No statistically significant difference in comorbidities and history of smoking and drinking was found (*p* > 0.05; Table 1).

**Table 1:** Baseline characteristics.

	**HUK group *(n* = 21)**	**HUK+Maixuekang group (*n* = 16)**	**Control group (*n* = 19)**	***P*-value**
**GENDER**				
Male, No. (%)	14 (66.67%)	11 (68.75)	13 (68.42%)	0.781
Female, No. (%)	7 (33.3%)	5 (31.25)	6 (31.58%)	0.692
Age, $\bar{\text{x}}$±s	58.10 ± 13.66	60.88 ± 11.85	61.1 ± 14.46	0.655
Smoking, No. (%)	12 (57.14%)	9 (56.25%)	11 (57.89%)	0.824
Drinking, No. (%)	9 (42.86%)	7 (43.75%)	8 (42.1%)	0.763
Hypertension, No. (%)	15 (71.42%)	11 (68.75%)	13 (68.4%)	0.543
Diabetes, No. (%)	6 (28.57%)	5 (31.25%)	6 (31.57%)	0.581
Atrial fibrillation	0	0	0	–
NIHSS score _beforetreatment_	5.19 ± 1.60	5.12 ± 1.75	5.21 ± 1.69	0.862
CHA_2_DS_2−_VAScscore	2.00 ± 1.19	2.13 ± 1.20	2.00+1.08	0.938
CRP_beforetreatment_	3.66 ± 1.05	3.17 ± 1.23	3.43 ± 1.07	0.845
**ECHOCARDIOGRAPHIC PARAMETERS**				
LA (mm)	33.69 ± 2.33	35.62 ± 3.59	34.55 ± 6.50	0.584
RA (mm)	35.89 ± 3.53	37.64 ± 5.33	36.69 ± 5.84	0.607
LV (mm)	49.31 ± 3.75	51.23 ± 4.82	50.64 ± 4.55	0.641
RV (mm)	16.75 ± 1.29	17.46 ± 2.44	17.36 ± 2.92	0.589
LVEF (%)	64.38 ± 7.93	64.85 ± 6.77	65.18 ± 6.70	0.665
E/A	0.95 ± 0.34	0.90 ± 0.53	0.83 ± 0.28	0.735

LA, left atrium dimension; RA, right atrium dimension; LV, left ventricle dimension; RV, right ventricle dimension; LVEF, left ventricular ejection fraction; E/A, E-point of septal separation/ A-point of septal separation. Hypertension was defined as systolic pressure ≥ 140 mmHg or diastolic pressure ≥ 90mmHg; diabetes was defined as a random blood glucose ≥ 200mg/dl or postprandial blood glucose ≥ 126 mg/dl; The clinical data of the three groups were compared, the ^*^P > 0.05, there were no statistical difference.

### Comparison of neurological deficiency in patients before and after treatment

NIHSS scores of control group were not significantly different before treatment (*P* > 0.05). NIHSS scores of the HUK group and HUK + Maixuekang group were significantly decreased after treatment (P _HUK_ = 0.001, P _HUK+Maixuekang_ < 0.001). NIHSS scores of the HUK group after treatment were significantly lower than that of the control group (*P* = 0.032). NIHSS scores of the HUK + Maixuekang group after treatment were significantly lower than that of the control group (*P* < 0.001). There were slight difference in NIHSS scores after treatment between the HUK group and the HUK + Maixuekang group (*P* = 0.068; Table 2).

**Table 2:** Comparison of NIHSS scores of three groups before and after treatment.

**Group**	NIHSS^*^ score before treatment	**NIHSS score 7days after treatment**	***P*-value**
HUK group (*n* = 21)	5.19 ± 1.60	3.33 ± 1.74^a^	0.001
HUK+Maixuekang group (*n* = 16)	5.12 ± 1.75	2.38 ± 1.20^b^	<0.001
Control group (*n* = 19)	5.21 ± 1.69	4.47 ± 1.47	0.159

* NIHSS, National Institutes of Health Stroke Scale;

a Comparison of the HUK group and control group, p = 0.032;

b *Comparison of the HUK + Maixuekang group and control group, p < 0.001*.

### Comparison of good functional outcome rates of three groups in 12 months after treatment

The good functional outcome rates of three groups in 12 month after treatment were significantly different (*P* = 0.031). The good functional outcome rates of the HUK group and control group were significantly different (*P* = 0.049). The good recovery rates of the HUK + Maixuekang group and control group were significantly different (*P* < 0.001). There were no significant difference in good recovery rates in 12 month after treatment between the HUK group and the HUK + Maixuekang group (*P* = 0.742). There were no significant difference in incidence of acute cerebral events among three groups (*P* = 0.091; Table 3).

**Table 3:** Comparison of good Functional Outcome (mRS score on) rates and incidence of acute cerebral events of three groups in 12 months after treatment.

**12 month mRS**	HUK group^a*^ (*n* = 21)	**HUK+Maix group^b^ (*n* = 16)**	**Control group (*n* = 19)**	***P***
Good outcome (mRS score ≤ 2)	9 (42.8%)	5 (31.2%)	14 (73.7%)	0.031
Cerebral events	2 (9.5%)	1 (6.2%)	2 (10.5%)	0.091

*a *Comparison of the HUK group and control group, p = 0.049*.

b *Comparison of the HUK + Maixuekang group and control group < 0.001*.

## Discussion

Nerve tissue necrosis after acute ischemic stroke is caused by the stenosis or occlusion of arteries or veins which leads to ischemia and hypoxia changes in the blood-supply area and eventually results in neurological deficit symptoms. Therapies like thrombolysis are essential to rescue ischemic tissue and alleviate neurologic impairment for AIS patients (Patel and Saver, 2013). In this study, HUK and Maixuekang capsule promoted favorable recovery in AIS patients, suggesting that HUK and Maixuekang capsule had the potential therapeutic value in the AIS patients by participating pathophysiology of acute ischemic stroke.

HUK is a kind of glycoprotein extracted from male urine that can produce kallikrein. Kallikrein, kiniogen, kinin, and its receptors are the most important components of KKS (Albert-Weißenberger et al., 2013). Since Abelous and Bardier first proposed that KKS has the effect of reducing blood pressure in dogs in 1909 (Abelous and Bardier, 1909), KKS system has gradually become the focus of studies. Kallikrein is a kind of serine protease which can enzymatically accelerate the production of kinin from kiniogen. The kinin act through its receptors, B1R and B2R (members of rhodopsin-class of GPCR superfamily), which mediate the multiple pathophysiological functions of kinin, including vascular permeability, edema formation, transendothelial cell migration, and inflammation following injury (Leeb-Lundberg et al., 2005). Numerous animal trials have proven that stroke can affect the presence of KKS components especially the kinin and its receptors. The B1R and B2R RNA and protein expression are increased after ischemic stroke and the kinin also increase in parallel with its receptor (Austinat et al., 2009).

The injection of HUK gene immediately after middle cerebral artery occlusion (MCAO) can inhibit apoptosis and inflammation, and induce revascularization and neurotization in rat (Xia et al., 2006). Multiple experimental studies have shown that the kinin receptors play an important role in the pathophysiology of acute ischemic stroke. The application of CP-0597, which is the specific B2R peptide antagonist, can reduce brain swelling, infarct size, and neuronal damage in rats after permanent cerebral ischemia (Relton et al., 1997). The specific non-peptide B2R antagonist LF 16-0687 (Anatibant) can also effectively reduce the inflammatory response, BBB disruption, and infarct growth, and had a neuroprotective effect after stroke (Zausinger et al., 2002). Further, some studies have suggested that the role of B1R is more important than B2R in the pathophysiology of acute ischemic stroke. There is an ~50% reduction in infarct size in B1r-deficient mice when compared with wild-type mice after stroke, and it is associated with an improved functional outcome (Austinat et al., 2009). The inflammation is reduced and there was nearly absent edema formation in B1r-deficient mice after ischemic stroke. Therefore, the KKS is implicated in multiple pathological states, and represents an attractive therapeutic target in acute ischemic stroke.

Maixuekang capsule is a Chinese medicine preparation extracted from freeze dried powder of fresh leech. The main component is hirudin, an active anticoagulant produced by leech salivary glands, and is considered to be the most effective and safe thrombin inhibitor (Whitaker et al., 2004). Some studies have shown that hyperfibrinogenemia is an independent risk factor of cerebrovascular atherosclerosis, and the increase of plasma fibrinogen is related to poor prognosis of ischemic cerebrovascular disease (Mauriello et al., 2000). The reduction of fibrinogen can effectively improve the incidence of poor prognosis in AIS patients (Sherman et al., 2000). Hirudin can rapidly inhibit blood coagulation through restraining cleavage of fibrinogen by binding with thrombin that improve the viscosity of blood and prevent the formation of thrombosis (Prisco et al., 2001). Oxidative stress and inflammation play an important role in the pathophysiology of acute ischemic stroke, the inflammatory biomarkers may be predictive of clinical outcomes (Sardu et al., 2017), patients in this study have no difference in the level of CRP, which is a common marker of inflammation. On the other hand, the anti-oxidative therapy may control the vascular events (Marfella et al., 2016; Sardu et al., 2017), and basic treatment including anti-oxidative therapy and other symptomatic treatment was given to all patients in this study.

From the results of this study, we not only proved that the treatment of HUK play an effective role in the recovery of AIS, but also found, for the first time, the treatment of HUK combined with Maixuekang capsule can significantly reduce the degree of neurologic impairment and improve clinical outcomes of patients after 12 month treatment. However, we didn't find significant difference between treatment of HUK combined with Maixuekang capsule and the treatment of HUK, neither in NIHSS scores after 7 days treatment nor in the good recovery rates after 12 month treatment, but we could see a trend that the NIHSS scores in combined treatment group decreased more significant than that in HUK group (*P* = 0.068). This might be caused by the small size of samples, which couldn't reflect the significant difference between different groups.

### Study limitation

(1) This is a single center study with small samples, which cannot be fully subgroup analyzed to prove the effect of diabetes and other factors, which may influence the prognosis of vascular brain disease (Marfella et al., 2013) on the outcome of stroke. (2) Besides, Although we excluded patients with severe infectious disease, we just recorded CRP level not other data of inflammatory and oxidative stress and the circulating serum levels of common clinical practice biomarkers (for example BNP), which may provide predictive value of clinical outcome (Katsanos et al., 2017). (3) Furthermore, in this study, we didn't enrolled patients who were treated with Maixuekang capsule alone, which maked it impossible to compare the efficacy of HUK and Maixuekang capsule. (4) In addition, there was no patient with atrial fibrillation and other significant arrhythmic burden in this study, and not every patient had continuous rhythm monitoring data and we did not collect that in this study which may provide useful information to predict clinical outcomes (Sardu et al., 2016), for example, heart rate variability may lead to autonomic dysfunction in a target population, and this may predict arrhythmic events leading to vascular brain disease (Rizzo et al., 2015), so we cannot be informed of the effort of therapies in this study on stroke patients with atrial fibrillation or other arrhythmic burden. Therefore, multi-center, large sample, double-blind, and randomized studies are needed to further validate the results of this study.

## Conclusion

In summary, the therapeutic strategy of HUK or HUK combined with Maixuekang capsule could improve favorable recovery in AIS patients. Our study provide a new therapy choice for patients with AIS. Whether the combined treatment is better than HUK treatment, further investigation is needed in future.

## Author contributions

JS and YL were responsible for the study design, cases selection, data extraction, and manuscript preparation. MW and JZ were responsible for data extraction. LG and XT were responsible for study design and manuscript revisions.

### Conflict of interest statement

The authors declare that the research was conducted in the absence of any commercial or financial relationships that could be construed as a potential conflict of interest.
